# Emerging roles of small GTPases in secondary cell wall development

**DOI:** 10.3389/fpls.2014.00428

**Published:** 2014-08-26

**Authors:** Yoshihisa Oda, Hiroo Fukuda

**Affiliations:** ^1^Center for Frontier Research, National Institute of GeneticsMishima, Japan; ^2^The Graduate University For Advanced StudiesMishima, Japan; ^3^Precursory Research for Embryonic Science and Technology, Japan Science and Technology AgencyKawaguchi, Japan; ^4^Department of Biological Sciences, Graduate School of Science, The University of TokyoTokyo, Japan

**Keywords:** secondary cell wall, ROP GTPase, Rab GTPase, cytoskeleton, xylem

## Abstract

Regulation of plant cell wall deposition and patterning is essential for the normal growth and development of plants. Small GTPases play pivotal roles in the modulation of primary cell wall formation by controlling cytoskeletal organization and membrane trafficking. However, the functions of small GTPases in secondary cell wall development are poorly understood. Recent studies on xylem cells revealed that the Rho of plants (ROP) group of small GTPases critically participates in the spatial patterning of secondary cell walls. In differentiating xylem cells, a specific GTPase-activating protein (GAP)/guanine nucleotide exchange factor (GEF) pair facilitates local activation of ROP11 to establish *de novo* plasma membrane domains. The activated ROP11 then recruits a microtubule-associated protein, MIDD1, to mediate the mutual inhibition between cortical microtubules and active ROP. Furthermore, recent works suggest that certain small GTPases, including ROP and Rab GTPases, regulate membrane trafficking to establish secondary cell wall deposition and patterning. Accordingly, this mini-review assesses and summarizes the current literature regarding the emerging functions of small GTPases in the development of secondary cell walls.

## INTRODUCTION

Post-embryonic development of plants requires prominent cell growth and cell differentiation phases, during which cells dramatically increase their volume and assume appropriate shapes and functions. The plant cell-shaping process depends entirely on the presence of a well-ordered cellulosic primary cell wall, which restricts cell enlargement to ensure anisotropic cell expansion in the direction perpendicular to the cellulose micro fibrils. In contrast to the relatively thin and elastic primary cell wall found in all plant cells, secondary cell walls are thick, lignified structures deposited between the primary cell wall and the plasma membrane, and are only found in mature and non-dividing cells in vascular plants. Massive secondary cell walls provide mechanical rigidity and strength to xylem components, including xylem fibers, xylem vessels, and xylem parenchyma cells. In this manner, secondary cell walls support the plant form and facilitate water transport throughout the plant (Ye et al., 2002). For example, the woody tissue of trees is mainly made of secondary cell walls developed in the secondary xylem (Demura and Ye, 2010). Secondary cell walls are also developed in the anther and silique pods (the two-valved seed pods of plants of the crucifier family), where they enable dehiscence of the anther and shattering of the pod, respectively (Mitsuda et al., 2005). Although the nature of the secondary cell wall differs from that of the primary cell wall, proper patterning of cell wall materials is similarly essential for the normal function of secondary cell walls (Zhong et al., 2002).

The deposition pattern of cellulose microfibrils in plant cell walls is largely contingent on the cortical microtubules that regulate the trajectory of cellulose synthase complexes during both primary and secondary cell wall development (McFarlane et al., 2014). Cortical microtubules act as a target site for the *trans*-Golgi network vesicles that deliver cellulose synthase complexes to cell wall deposition locales (Crowell et al., 2009; Gutierrez et al., 2009). Polarized actin microfilaments are also critical for the secretion of the membrane compartments that supply cellulose synthases and other cell wall synthetic materials to the apex of tip-growing root hairs and pollen tubes, and the consequent maintenance of polarized tip growth (Park et al., 2011; Gu and Nielsen, 2013; Rounds and Bezanilla, 2013). In general, cytoskeletal organization and membrane trafficking are essential for the proper formation of primary and secondary cell walls.

Rho and Rab family small GTPases are crucial for the regulation of these processes in higher plants. Rab GTPases mainly control the targeting and tethering of membrane compartments, while Rho GTPases mainly control the polarized organization of the cytoskeleton and vesicle trafficking, as well as various cellular responses against biotic/abiotic stress and other events. Plants have evolved a Rho of plants (ROP) family of GTPases as a counterpart to the families of conserved Rho/Rac GTPases in animal and yeast cells (Nagawa et al., 2010). ROP GTPases, Rab GTPases, and other small GTPases typically bind to GTP and then interact with effector proteins, thereby stimulating relevant signaling cascades in the plant cell. The GTP-bound active form of the small GTPase is inactivated by GTPase-activating proteins (GAPs), which promote hydrolysis of GTP. In contrast, guanine nucleotide exchange factors (GEFs) catalyze the activation of small GTPases by exchanging GTP for GDP. Thus, GAPs and GEFs ensure spatially and temporally controlled action of Rho and Rab GTPases to modulate plant cell cytoskeletal organization and membrane trafficking.

Rho of plants and Rab GTPases play important roles in the patterning and deposition of primary cell walls. Rab GTPases regulate the delivery of cell wall synthetic materials and secreted proteins to the growing apex via actin microfilaments in growing root hairs and pollen tubes (de Graaf et al., 2005; Szumlanski and Nielsen, 2009), whereas ROP GTPases coordinate the actions of cortical microtubules, actin microfilaments, and membrane trafficking in growing pollen tubes, root hairs, and epidermal cells (Yang, 2008; Craddock et al., 2012). However, the roles of small GTPases in secondary cell wall formation are still poorly understood, although a few studies have implicated the presence of ROP GTPases in xylem cells (Nakanomyo et al., 2002; Brembu et al., 2005; Ko et al., 2006).

Recent studies on xylem vessel cells revealed that ROP GTPases are central to the spatial patterning of secondary cell walls. Because xylem vessels exhibit distinct patterns of secondary cell wall deposition (i.e., annular, spiral, reticulate, scaraliform, and pitted patterns), investigations of xylem vessels provide an effective platform to explore the molecular mechanisms underlying the spatial control of secondary cell wall deposition. The purpose of this mini-review is thus to summarize recent progress toward elucidation of the roles of small GTPases in secondary cell wall development in the xylem of higher plants.

## ROP GTPase SIGNALING INITIATES PATTERNING OF THE SECONDARY CELL WALL

Metaxylem vessels form secondary cell walls with numerous secondary cell wall pits in cases where the secondary cell wall is not deposited for lateral transport of xylem sap. Oda and Fukuda (2012a) reported that ROP signaling initiates the spatial patterning of secondary cell wall deposition in metaxylem vessels, and that the ROP11 GTPase is locally activated in secondary cell wall pits in *Arabidopsis thaliana* metaxylem cells. Introduction of a constitutively activate form of ROP11 into differentiating xylem cells inhibited the typical patterning of secondary cell walls, instead resulting in the formation of a flat secondary cell wall lacking secondary wall pits. These observations demonstrate that limited ROP activation is required for secondary cell wall patterning.

The authors also found that ROPGEF4 (an ROP GEF) and ROPGAP3 (an ROP GAP) are co-localized in secondary cell wall pits, and that loss of ROPGEF4 leads to a reduced number of secondary cell wall pits. Importantly, co-introduction of ROPGEF4, ROPGAP3, and ROP11 into non-xylem cells resulted in the presence of disperse spots of active ROP11 on the plasma membrane in a manner reminiscent of the localization of active ROP11 in xylem cells. These findings demonstrate that ROPGEF4 and ROPGAP3 mediate *de novo* local activation of ROP11 to establish the fundamental patterning of secondary cell wall pits (Oda and Fukuda, 2012a). While the precise mechanism by which ROPGEF4 and ROPGAP3 organize active ROP11 into discrete patches on the plasma membrane remains unclear, these effector molecules are undoubtedly indispensable to the regulatory machinery that determines secondary cell wall pit positioning and density.

## ROP11 SIGNALING LOCALLY INHIBITS SECONDARY CELL WALL DEPOSITION

Cortical microtubules are arranged into distinct patterns that direct secondary cell wall deposition (Oda et al., 2005; Oda and Hasezawa, 2006). Various microtubule-associated proteins reportedly regulate cortical microtubule rearrangements during xylem vessel differentiation (Mao et al., 2006; Pesquet et al., 2010; Oda and Fukuda, 2012b, 2013c). Recent work showed that the rearrangement process proceeds through the local destabilization of cortical microtubules under the control of the plant-specific microtubule-associated protein, MIDD1, and the microtubule depolymerizer, kinesin-13A (Oda et al., 2010; Oda and Fukuda, 2013b).

MIDD1 belongs to the ROP-binding family of ROP interactive partner (RIP)/interactor of constitutive active ROPs (ICRs) proteins (Lavy et al., 2007; Li et al., 2008). MIDD1 is preferentially expressed in differentiating xylem cells (Oda et al., 2010). The ROP-binding protein is anchored to the plasma membrane in secondary cell wall pits by activated ROP11, where it destabilizes cortical microtubules. Knockdown of MIDD1 inhibits local disassembly of cortical microtubules, resulting in the loss of secondary cell wall pits (Oda et al., 2010). Nevertheless, MIDD1 has no activity to depolymerize cortical microtubules by itself, but instead recruits kinesin-13A to provoke microtubule depolymerization (Oda and Fukuda, 2013b). On the other hand, kinesin-13A alone has microtubule depolymerization activity *in vitro*, but requires positioning of MIDD1 at cortical microtubule sites to exert its depolymerization activity *in vivo*. Loss and overexpression of kinesin-13A leads to the formation of smaller than normal or larger than normal secondary cell wall pits, respectively, suggesting that ROP11 signaling locally inhibits secondary cell wall deposition through the disassembly of cortical microtubules via the MIDD1/kinesin-13A complex (Oda and Fukuda, 2013b).

## ROP11 SIGNALING MODULATES GEOMETRY OF THE SECONDARY CELL WALL

Microtubule stabilization by taxol causes formation of elongated, oblique secondary cell wall pits in xylem vessels (Falconer and Seagull, 1985; Oda et al., 2005). Therefore, cortical microtubules are apparently not the only target of ROP11/MIDD1/kinesin-13A signaling. Rather, cortical microtubules might act to counteract ROP11 signaling under certain circumstances. Oda and Fukuda (2012a) demonstrated this concept by reconstructing the plasma membrane domains of active ROP11 in the non-xylem leaf epidermis. The cortical microtubules restricted the border of the reconstructed, active ROP11 domains and maintained them in an oval shape, whereas microtubule disruption deformed the domains into a round shape (Oda and Fukuda, 2012a, 2013a). The microtubule-mediated restriction of active ROP11 domains also required full-length MIDD1. In xylem cells, disruption of the cortical microtubules yielded diffusion of the active ROP GTPase away from the secondary cell wall pits (Oda and Fukuda, 2012a, 2013a). Therefore, ROP11 signaling appears to act as a spatial buffer between cortical microtubule depolymerization and polymerization sites by facilitating a mutual inhibition between microtubule assembly and the ROP11/MIDD1/kinesin-13A pathway.

Global cortical microtubule polymerization activity may affect ROP-mediated spatial buffering to influence the formation of various secondary cell wall patterns, where higher microtubule polymerization activity is expected to strongly restrict the area of depolymerization and to enhance the formation of secondary cell walls with smaller and narrower secondary wall pits. By contrast, weaker microtubule polymerization activity is expected to be less restrictive and to enhance the formation of secondary cell walls with larger and rounder secondary cell wall pits. Therefore, this regulatory system might account for the assorted deposition patterns of secondary cell walls.

## ROP GTPases MAY FUNCTION IN DIRECTED EXOCYTOSIS DURING PATTERNED SECONDARY CELL WALL DEPOSITION

Considering the remarkable deposition of secondary walls in xylem cells, it is reasonable to hypothesize that strictly directed exocytosis takes place during xylem differentiation to supply various cell wall synthetic materials to the developing walls. Octameric complexes termed exocysts mediate membrane fusion during directed exocytosis and are composed of eight conserved subunits: subunit of exocyst complex (SEC) 3, SEC5, SEC6, SEC8, SEC10, SEC15, EXOcyst (EXO) 70, and EXO84 (Hala et al., 2008). In yeast, SEC3 and EXO70 are localized at the target membrane of *trans*-Golgi network vesicles, while the other exocyst complex members are localized at the vesicle membrane itself.

Li et al. (2010, 2013) revealed that an exocyst subunit gene, *EXO70A1* (one of 23 EXO70 genes encoded in the *Arabidopsis* genome) is preferentially expressed in xylem cells and is required for normal secondary cell wall development. Loss of *EXO70A1* leads to disorganized secondary cell wall thickening and abnormal accumulation of membrane compartments in differentiating xylem cells, with no inhibition of secondary cell wall deposition. This finding indicates that EXO70A1 regulates the targeting of exocytotic vesicles to the cortical microtubule-marked plasma membrane in differentiating xylem cells.

Rho GTPases directly bind to EXO70 and SEC3 subunits in yeast and mammalian cells and target exocytotic vesicles to particular plasma membrane domains (Robinson et al., 1999; He and Guo, 2009; Wu et al., 2010). Several lines of evidence suggest that ROP GTPases regulate targeting of exocyst complexes in plants. For example, a number of exocyst subunits, including EXO70A1, are localized at the growing tips of pollen tubes. Active ROP GTPases are also present at the growing tips of pollen tubes and root hairs (Cole et al., 2005; Hala et al., 2008) and are essential for polarized tip growth. Conversely, exocyst subunit mutants exhibit pollen tube and root hair defects (Wen et al., 2005; Synek et al., 2006). Importantly, SEC3 interacts with ROP-binding ICR1/RIP1 proteins in pollen tubes; like ROP GTPases, these interactions are essential for polarized pollen tube growth (Lavy et al., 2007), implying that ROP GTPases indirectly interact with the exocyst complex to modulate targeting of exocytotic vesicles. Similarly, ROP GTPases may regulate the localization of exocyst complexes in xylem cells to target exocytotic vesicles to cortical microtubule-tagged plasma membrane domains.

Additional evidence suggests that ROP GTPases are involved in secondary cell wall development. Foucart et al. (2009) reported that a *Eucalyptus* ROP GTPase gene, *EgROP1*, is strongly expressed in the cambium and differentiating xylem cells. Overexpression of constitutively activate EgROP1 in *Arabidopsis* inhibits lignification and xylan deposition in the secondary xylem, indicating that EgROP1 participates in secondary cell wall deposition (Foucart et al., 2009).

## Rabg3B GTPase PROMOTES SECONDARY CELL WALL DEPOSITION

Several studies suggest that a Rab GTPase contributes to the control of secondary cell wall deposition in higher plants. Kwon et al. (2010a) report that RabG3b is upregulated during xylem differentiation and is likely involved in secondary cell wall development. RabG3b is highly similar to mammalian Rab7 and yeast Ypt7, which direct the later steps of membrane trafficking during autophagy (Kirisako et al., 1999; Gutierrez et al., 2004; Jager et al., 2004). Kwon et al. (2010a,b, 2011) found that the expression of constitutively active RabG3b promoted autophagy and xylem cell development in *Arabidopsis* and *Populus*. This also occurred in cultured cells, where treatment with brassinosteroid and boric acid induced xylem cell differentiation *in vitro*. By contrast, knockdown of RabG3b or introduction of a dominant negative form of RabG3b impaired both autophagy and differentiation, whereas activation of RabG3b upregulated the expression of programmed cell death (PCD)-related genes and secondary wall syntheses-related genes. However, RabG3b activation did not upregulate genes involved in the induction of xylem cell differentiation. Therefore, RabG3b activation appears to specifically promote PCD and secondary cell wall development through autophagy, but does not promote the induction of xylem cell differentiation (Kwon et al., 2010a). Nevertheless, the precise mechanism whereby RabG3b induces secondary cell wall development is still uncertain and requires further exploration.

## SIGNALING THROUGH MULTIPLE ROP GTPases MAY SPATIALLY COORDINATE PROMOTION AND INHIBITION OF SECONDARY CELL WALL DEVELOPMENT

**FIGURE 1:**
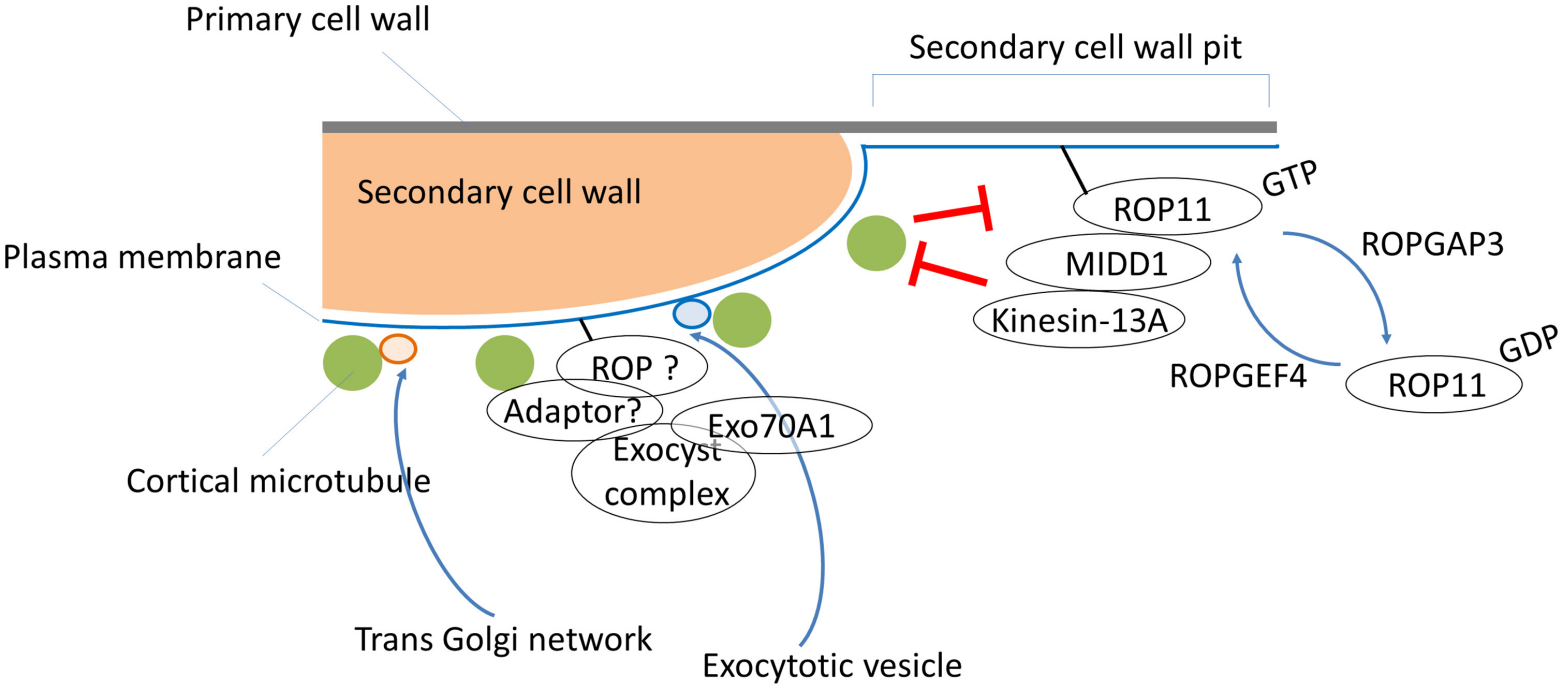
**A multi-Rho of plants (ROP) signaling model for secondary cell wall development.** ROPGEF4 and ROPGAP3 establish the ROP11/MIDD1/kinesin-13A pathway to generate a cortical microtubule-absent area by promoting microtubule depolymerization, which results in pits of secondary cell walls in metaxylem vessels. Cortical microtubules restrict signaling from the ROP11/MIDD1/kinesin-13A pathway and promote local secondary cell wall deposition via an unknown ROP pathway and/or an ROP-independent pathway that controls targeting of exocytotic vesicles through EXO70A1. An adaptor protein such as RIP1/ICR1 might mediate interactions between the putative ROP, exocysts, and cortical microtubules. Vesicles from the *trans*-Golgi network are directly targeted to cortical microtubules, and subsequently supply cellulose synthase complexes to the cellular sites where microtubules are present.

The *Arabidopsis* genome encodes 11 ROPs with divergent roles in microtubule and actin organization, membrane trafficking and the establishment of polarity, and responses to environmental and pathogenic cues (Nibau et al., 2006; Yang and Fu, 2007). Expression data indicate that at least eight ROPs including ROP11 are expressed in xylem cells (Winter et al., 2007).Because ROP11 functions in ROP/MIDD1/kinesin-13A signaling to form secondary cell wall pits, ROPs other than ROP11 may participate in secondary cell wall development. In this regard, two spatially discrete ROP GTPase signaling pathways act together in leaf epidermal cells to achieve interdigitated cell morphology: ROP6 signaling stabilizes cortical microtubules through the RIC1 effector, while ROP2 signaling stabilizes actin microfilaments through the RIC4 effector (Fu et al., 2005). These two signaling pathways mutually inhibit each other and enhance themselves through a positive feedback mechanism (Fu et al., 2005; Xu et al., 2010). Similarly, the plural ROP signaling pathways might exist in xylem cells to control cytoskeletal organization, which in turn regulates the targeting of exocytotic vesicles through EXO70A1 to the area where cortical microtubules are present (**Figure 1**). Another scenario, however, is still possible in which only the ROP11 pathway operates in xylem cells, and the exocytotic vesicles are targeted to the microtubule-positive area independently of the ROP GTPases. Because active ROP11 inhibits membrane recycling (Bloch et al., 2005), locally activated ROP11 may downregulate membrane recycling in the area where cortical microtubules are absent, thereby facilitating membrane trafficking outside the realm of active ROP11 domains where cortical microtubules are present. Alternatively, microtubules may function as a direct target of exocytosis. Further studies are required to elucidate the precise actions of ROP GTPases in directed exocytosis, and to uncover the regulatory mechanism of membrane trafficking in secondary cell wall development.

## PERSPECTIVES AND CONCLUDING STATEMENTS

Several studies on xylem cells have revealed that ROP and Rab GTPases essentially direct cortical microtubule organization and membrane trafficking to establish secondary cell wall patterning and deposition. In addition, targeting of exocytotic vesicles to the plasma membrane domains where cortical microtubules are aligned is likely under the control of ROP GTPase signaling. To understand the molecular processes behind secondary cell wall development, a number of issues must still be resolved. For example, secondary cell wall pits are usually generated in a face-to-face fashion, forming a pit pair that bridges two adjacent xylem cells. Recently, Xu et al. (2014) reported that transmembrane receptor-like kinases mediate intercellular communication through auxin and ROP GTPase signaling during pavement cell morphogenesis (Xu et al., 2014). Furthermore, certain ROP GEFs interact with receptor-like kinases (Duan et al., 2010; Akamatsu et al., 2013; Chang et al., 2013; Huang et al., 2013). These observations raise the hypothesis that intercellular signaling might regulate the position of secondary cell wall pit pairs via the ROP11/MIDD1/kinesin-13A pathway.

Another issue concerns the question of whether ROP GTPase signaling is involved in secondary cell wall patterning in protoxylem cells, where secondary cell walls are deposited in annular or spiral patterns. Interestingly, overexpression or knockdown of signaling molecules in the ROP11/MIDD1/kinesin-13A pathway only slightly affected secondary cell wall formation in protoxylem vessels (Oda and Fukuda, 2012a, 2013b). These data suggest that discrete ROP signaling either does not crosstalk with the ROP11/MIDD1/kinesin-13A pathway, or that ROP-independent signaling may organize the secondary cell wall in protoxylem cells.

In addition, the manner in which small GTPases modulate membrane trafficking to ensure the local deposition of secondary cell walls at the area marked with cortical microtubules is an important issue for further exploration. Lastly, actin microfilaments are essential structural components of membrane trafficking in xylem cells (Wightman and Turner, 2008), and it would be most interesting to investigate the potential participation of small GTPases in the regulation of actin microfilaments in xylem cells. Future studies focusing on the functions of small GTPases will most certainly contribute to our further understanding of regulation of complicated secondary cell wall development.

## Conflict of Interest Statement

The Guest Associate Editor Takashi Ueda declares that, despite being affiliated to the same institution as author Hiroo Fukuda, the review process was handled objectively and no conflict of interest exists. The authors declare that the research was conducted in the absence of any commercial or financial relationships that could be construed as a potential conflict of interest.

## References

[B1] Akamatsu A. Wong H. L. Fujiwara M. Okuda J. Nishide K. Uno K. (2013). An OsCEBiP/OsCERK1-OsRacGEF1-OsRac1 module is an essential early component of chitin-induced rice immunity. *Cell Host Microbe* 13 465–476 10.1016/j.chom.2013.03.007 23601108

[B2] Bloch D. Lavy M. Efrat Y. Efroni I. Bracha-Drori K. Abu-Abied M. (2005). Ectopic expression of an activated RAC in *Arabidopsis* disrupts membrane cycling. *Mol. Biol. Cell* 16 1913–1927 10.1091/mbc.E04-07-0562 15703216 PMC1073671

[B3] Brembu T. Winge P. Bones A. M. (2005). The small GTPase AtRAC2/ROP7 is specifically expressed during late stages of xylem differentiation in *Arabidopsis*. *J. Exp. Bot.* 56 2465–2476 10.1093/jxb/eri239 16061508

[B4] Chang F. Gu Y. Ma H. Yang Z. (2013). AtPRK2 promotes ROP1 activation via RopGEFs in the control of polarized pollen tube growth. *Mol. Plant* 6 1187–1201 10.1093/mp/sss103 23024212 PMC3888354

[B5] Cole R. A. Synek L. Zarsky V. Fowler J. E. (2005). SEC8, a subunit of the putative *Arabidopsis* exocyst complex, facilitates pollen germination and competitive pollen tube growth. *Plant Physiol.* 138 2005–2018 10.1104/pp.105.062273 16040664 PMC1183391

[B6] Craddock C. Lavagi I. Yang Z. (2012). New insights into Rho signaling from plant ROP/Rac GTPases. *Trends Cell Biol.* 22 492–501 10.1016/j.tcb.2012.05.002 22795444 PMC3432703

[B7] Crowell E. F. Bischoff V. Desprez T. Rolland A. Stierhof Y. D. Schumacher K. (2009). Pausing of Golgi bodies on microtubules regulates secretion of cellulose synthase complexes in *Arabidopsis*. *Plant Cell* 21 1141–1154 10.1105/tpc.108.065334 19376932 PMC2685615

[B8] de Graaf B. H. Cheung A. Y. Andreyeva T. Levasseur K. Kieliszewski M. Wu H. M. (2005). Rab11 GTPase-regulated membrane trafficking is crucial for tip-focused pollen tube growth in tobacco. *Plant Cell* 17 2564–2579 10.1105/tpc.105.033183 16100336 PMC1197435

[B9] Demura T. Ye Z. H. (2010). Regulation of plant biomass production. *Curr. Opin. Plant Biol.* 13 299–304 10.1016/j.pbi.2010.03.002 20381410

[B10] Duan Q. Kita D. Li C. Cheung A. Y. Wu H. M. (2010). FERONIA receptor-like kinase regulates RHO GTPase signaling of root hair development. *Proc. Natl. Acad. Sci. U.S.A.* 107 17821–17826 10.1073/pnas.1005366107 20876100 PMC2955125

[B11] Falconer M. M. Seagull R. W. (1985). Xylogenesis in tissue-culture - taxol effects on microtubule reorientation and lateral association in differentiating cells. *Protoplasma* 128 157–166 10.1007/Bf01276337

[B12] Foucart C. Jauneau A. Gion J. M. Amelot N. Martinez Y. Panegos P. (2009). Overexpression of EgROP1, a Eucalyptus vascular-expressed Rac-like small GTPase, affects secondary xylem formation in *Arabidopsis thaliana*. *New Phytol.* 183 1014–1029 10.1111/j.1469-8137.2009.02910.x 19549133

[B13] Fu Y. Gu Y. Zheng Z. Wasteneys G. Yang Z. (2005). *Arabidopsis* interdigitating cell growth requires two antagonistic pathways with opposing action on cell morphogenesis. *Cell* 120 687–700 10.1016/j.cell.2004.12.026 15766531

[B14] Gu F. Nielsen E. (2013). Targeting and regulation of cell wall synthesis during tip growth in plants. *J. Integr. Plant Biol.* 55 835–846 10.1111/jipb.12077 23758901

[B15] Gutierrez M. G. Munafo D. B. Beron W. Colombo M. I. (2004). Rab7 is required for the normal progression of the autophagic pathway in mammalian cells. *J. Cell Sci.* 117 2687–2697 10.1242/jcs.01114 15138286

[B16] Gutierrez R. Lindeboom J. J. Paredez A. R. Emons A. M. Ehrhardt D. W. (2009). *Arabidopsis* cortical microtubules position cellulose synthase delivery to the plasma membrane and interact with cellulose synthase trafficking compartments. *Nat. Cell Biol.* 11 797–806 10.1038/ncb1886 19525940

[B17] Hala M. Cole R. Synek L. Drdova E. Pecenkova T. Nordheim A. (2008). An exocyst complex functions in plant cell growth in *Arabidopsis* and tobacco. *Plant Cell* 20 1330–1345 10.1105/tpc.108.059105 18492870 PMC2438459

[B18] He B. Guo W. (2009). The exocyst complex in polarized exocytosis. *Curr. Opin. Cell Biol.* 21 537–542 10.1016/j.ceb.2009.04.007 19473826 PMC2725219

[B19] Huang G. Q. Li E. Ge F. R. Li S. Wang Q. Zhang C. Q. (2013). Arabidopsis RopGEF4 and RopGEF10 are important for FERONIA-mediated developmental but not environmental regulation of root hair growth. *New Phytol.* 200 1089–1101 10.1111/nph.12432 23915272

[B20] Jager S. Bucci C. Tanida I. Ueno T. Kominami E. Saftig P. (2004). Role for Rab7 in maturation of late autophagic vacuoles. *J. Cell Sci.* 117 4837–4848 10.1242/jcs.01370 15340014

[B21] Kirisako T. Baba M. Ishihara N. Miyazawa K. Ohsumi M. Yoshimori T. (1999). Formation process of autophagosome is traced with Apg8/Aut7p in yeast. *J. Cell Biol.* 147 435–446 10.1083/jcb.147.2.435 10525546 PMC2174223

[B22] Ko J. H. Beers E. P. Han K. H. (2006). Global comparative transcriptome analysis identifies gene network regulating secondary xylem development in *Arabidopsis thaliana*. *Mol. Genet. Genomics* 276 517–531 10.1007/s00438-006-0157-1 16969662

[B23] Kwon S. I. Cho H. J. Jung J. H. Yoshimoto K. Shirasu K. Park O. K. (2010a). The Rab GTPase RabG3b functions in autophagy and contributes to tracheary element differentiation in *Arabidopsis*. *Plant J.* 64 151–164 10.1111/j.1365-313X.2010.04315.x 20659276

[B24] Kwon S. I. Cho H. J. Park O. K. (2010b). Role of *Arabidopsis* RabG3b and autophagy in tracheary element differentiation. *Autophagy* 6 1187–1189 10.4161/auto.6.8.13429 20861670

[B25] Kwon S. I. Cho H. J. Lee J. S. Jin H. Shin S. J. Kwon M. (2011). Overexpression of constitutively active *Arabidopsis* RabG3b promotes xylem development in transgenic poplars. *Plant Cell Environ.* 34 2212–2224 10.1111/j.1365-3040.2011.02416.x 21895694

[B26] Lavy M. Bloch D. Hazak O. Gutman I. Poraty L. Sorek N. (2007). A Novel ROP/RAC effector links cell polarity, root-meristem maintenance, and vesicle trafficking. *Curr. Biol.* 17 947–952 10.1016/j.cub.2007.04.038 17493810

[B27] Li S. Chen M. Yu D. Ren S. Sun S. Liu L. (2013). EXO70A1-mediated vesicle trafficking is critical for tracheary element development in *Arabidopsis*. *Plant Cell* 25 1774–1786 10.1105/tpc.113.112144 23709627 PMC3694705

[B28] Li S. Gu Y. Yan A. Lord E. Yang Z. B. (2008). RIP1 (ROP Interactive Partner 1)/ICR1 marks pollen germination sites and may act in the ROP1 pathway in the control of polarized pollen growth. *Mol. Plant* 1 1021–1035 10.1093/mp/ssn051 19825600 PMC9345201

[B29] Li S. Van Os G. M. Ren S. Yu D. Ketelaar T. Emons A. M. (2010). Expression and functional analyses of EXO70 genes in *Arabidopsis* implicate their roles in regulating cell type-specific exocytosis. *Plant Physiol.* 154 1819–1830 10.1104/pp.110.164178 20943851 PMC2996038

[B30] Mao G. Buschmann H. Doonan J. H. Lloyd C. W. (2006). The role of MAP65-1 in microtubule bundling during Zinnia tracheary element formation. *J. Cell Sci.* 119 753–758 10.1242/jcs.02813 16449317

[B31] McFarlane H. E. Doring A. Persson S. (2014). The cell biology of cellulose synthesis. *Annu. Rev. Plant Biol.* 65 69–94 10.1146/annurev-arplant-050213-040240 24579997

[B32] Mitsuda N. Seki M. Shinozaki K. Ohme-Takagi M. (2005). The NAC transcription factors NST1 and NST2 of *Arabidopsis* regulate secondary wall thickenings and are required for anther dehiscence. *Plant Cell* 17 2993–3006 10.1105/tpc.105.036004 16214898 PMC1276025

[B33] Nagawa S. Xu T. Yang Z. (2010). RHO GTPase in plants: conservation and invention of regulators and effectors. *Small GTPases* 1 78–88 10.4161/sgtp.1.2.14544 21686259 PMC3116591

[B34] Nakanomyo I. Kost B. Chua N. H. Fukuda H. (2002). Preferential and asymmetrical accumulation of a Rac small GTPase mRNA in differentiating xylem cells of Zinnia elegans. *Plant Cell Physiol.* 43 1484–1492 10.1093/pcp/pcf170 12514245

[B35] Nibau C. Wu H. M. Cheung A. Y. (2006). RAC/ROP GTPases: ‘hubs’ for signal integration and diversification in plants. *Trends Plant Sci.* 11 309–315 10.1016/j.tplants.2006.04.003 16737841

[B36] Oda Y. Fukuda H. (2012a). Initiation of cell wall pattern by a Rho- and microtubule-driven symmetry breaking. *Science* 337 1333–1336 10.1126/science.1222597 22984069

[B37] Oda Y. Fukuda H. (2012b). Secondary cell wall patterning during xylem differentiation. *Curr. Opin. Plant Biol.* 15 38–44 10.1016/j.pbi.2011.10.005 22078063

[B38] Oda Y. Fukuda H. (2013a). The dynamic interplay of plasma membrane domains and cortical microtubules in secondary cell wall patterning. *Front. Plant Sci.* 4:511 10.3389/fpls.2013.00511 PMC3865431 24381577

[B39] Oda Y. Fukuda H. (2013b). Rho of plant GTPase signaling regulates the behavior of *Arabidopsis* kinesin-13A to establish secondary cell wall patterns. *Plant Cell* 25 4439–4450 10.1105/tpc.113.117853 24280391 PMC3875728

[B40] Oda Y. Fukuda H. (2013c). Spatial organization of xylem cell walls by ROP GTPases and microtubule-associated proteins. *Curr. Opin. Plant Biol.* 16 743–748 10.1016/j.pbi.2013.10.010 24210792

[B41] Oda Y. Hasezawa S. (2006). Cytoskeletal organization during xylem cell differentiation. *J. Plant Res.* 119 167–177 10.1007/s10265-006-0260-8 16570127

[B42] Oda Y. Iida Y. Kondo Y. Fukuda H. (2010). Wood cell-wall structure requires local 2D-microtubule disassembly by a novel plasma membrane-anchored protein. *Curr. Biol.* 20 1197–1202 10.1016/j.cub.2010.05.038 20619818

[B43] Oda Y. Mimura T. Hasezawa S. (2005). Regulation of secondary cell wall development by cortical microtubules during tracheary element differentiation in *Arabidopsis* cell suspensions. *Plant Physiol.* 137 1027–1036 10.1104/pp.104.052613 15709154 PMC1065403

[B44] Park S. Szumlanski A. L. Gu F. Guo F. Nielsen E. (2011). A role for CSLD3 during cell-wall synthesis in apical plasma membranes of tip-growing root-hair cells. *Nat. Cell Biol.* 13 973–980 10.1038/ncb2294 21765420

[B45] Pesquet E. Korolev A. V. Calder G. Lloyd C. W. (2010). The microtubule-associated protein AtMAP70-5 regulates secondary wall patterning in *Arabidopsis* wood cells. *Curr. Biol.* 20 744–749 10.1016/j.cub.2010.02.057 20399097

[B46] Robinson N. G. Guo L. Imai J. Toh E. A. Matsui Y. Tamanoi F. (1999). Rho3 of *Saccharomyces cerevisiae*, which regulates the actin cytoskeleton and exocytosis, is a GTPase which interacts with Myo2 and Exo70. *Mol. Cell. Biol.* 19 3580–3587 10207081 10.1128/mcb.19.5.3580 PMC84150

[B47] Rounds C. M. Bezanilla M. (2013). Growth mechanisms in tip-growing plant cells. *Annu. Rev. Plant Biol.* 64 243–265 10.1146/annurev-arplant-050312-120150 23451782

[B48] Synek L. Schlager N. Elias M. Quentin M. Hauser M. T. Zarsky V. (2006). AtEXO70A1, a member of a family of putative exocyst subunits specifically expanded in land plants, is important for polar growth and plant development. *Plant J.* 48 54–72 10.1111/j.1365-313X.2006.02854.x 16942608 PMC2865999

[B49] Szumlanski A. L. Nielsen E. (2009). The Rab GTPase RabA4d regulates pollen tube tip growth in *Arabidopsis thaliana*. *Plant Cell* 21 526–544 10.1105/tpc.108.060277 19208902 PMC2660625

[B50] Wen T. J. Hochholdinger F. Sauer M. Bruce W. Schnable P. S. (2005). The roothairless1 gene of maize encodes a homolog of sec3, which is involved in polar exocytosis. *Plant Physiol.* 138 1637–1643 10.1104/pp.105.062174 15980192 PMC1176433

[B51] Wightman R. Turner S. R. (2008). The roles of the cytoskeleton during cellulose deposition at the secondary cell wall. *Plant J.* 54 794–805 10.1111/j.1365-313X.2008.03444.x 18266917

[B52] Winter D. Vinegar B. Nahal H. Ammar R. Wilson G. V. Provart N. J. (2007). An “Electronic Fluorescent Pictograph” browser for exploring and analyzing large-scale biological data sets. *PLoS ONE* 2:e718 10.1371/journal.pone.0000718 PMC1934936 17684564

[B53] Wu H. Turner C. Gardner J. Temple B. Brennwald P. (2010). The Exo70 subunit of the exocyst is an effector for both Cdc42 and Rho3 function in polarized exocytosis. *Mol. Biol. Cell* 21 430–442 10.1091/mbc.E09-06-0501 19955214 PMC2814788

[B54] Xu T. Dai N. Chen J. Nagawa S. Cao M. Li H. (2014). Cell surface ABP1-TMK auxin-sensing complex activates ROP GTPase signaling. *Science* 343 1025–1028 10.1126/science.1245125 24578577 PMC4166562

[B55] Xu T. Wen M. Nagawa S. Fu Y. Chen J. G. Wu M. J. (2010). Cell surface- and rho GTPase-based auxin signaling controls cellular interdigitation in *Arabidopsis*. *Cell* 143 99–110 10.1016/j.cell.2010.09.003 20887895 PMC2950838

[B56] Yang Z. (2008). Cell polarity signaling in *Arabidopsis*. *Annu. Rev. Cell Dev. Biol.* 24 551–575 10.1146/annurev.cellbio.23.090506.123233 18837672 PMC2739732

[B57] Yang Z. Fu Y. (2007). ROP/RAC GTPase signaling. *Curr. Opin. Plant Biol.* 10 490–494 10.1016/j.pbi.2007.07.005 17709276 PMC2956068

[B58] Ye Z. H. Freshour G. Hahn M. G. Burk D. H. Zhong R. (2002). Vascular development in *Arabidopsis*. *Int. Rev. Cytol.* 220 225–256 10.1016/S0074-7696(02)20007-8 12224550

[B59] Zarsky V. Kulich I. Fendrych M. Pecenkova T. (2013). Exocyst complexes multiple functions in plant cells secretory pathways. *Curr. Opin. Plant Biol.* 16 726-733. 10.1016/j.pbi.2013.10.013. 24246229

[B60] Zhong R. Q. Burk D. H. Morrison W. H. Ye Z. H. (2002). A kinesin-like protein is essential for oriented deposition of cellulose microfibrils and cell wall strength. *Plant Cell* 14 3101–3117 10.1105/Tpc.005801 12468730 PMC151205

